# Effects of climate variables on the incidence of scorpion stings in Iran for five years

**DOI:** 10.1590/1678-9199-JVATITD-2020-0110

**Published:** 2021-06-30

**Authors:** Ahmad Ghorbani, Behzad Mansouri, Masoumeh Baradaran

**Affiliations:** 1 Toxicology Research Center, Medical Basic Sciences Institute, Ahvaz Jundishapur University of Medical Sciences, Ahvaz, Iran. Toxicology Research Center Medical Basic Sciences Institute Ahvaz Jundishapur University of Medical Sciences Ahvaz Iran; 2 Department of Statistics, Shahid Chamran University of Ahvaz, Ahvaz, Iran. Department of Statistics Shahid Chamran University of Ahvaz Ahvaz Iran

**Keywords:** Scorpion stings, Climate factors, Scorpionism, Khuzestan province, Iran

## Abstract

**Background:**

Although scorpionism is recorded worldwide, some regions such as Iran present a higher incidence. Due to the great prevalence of scorpion stings in Khuzestan province, southwestern Iran, the present study examined the relationship between different climate parameters and the scorpion sting rate in this area from April 2010 to March 2015.

**Methods:**

In this cross-sectional descriptive-analytical study, we considered all scorpion sting cases recorded in the Department of Infectious Diseases, Ahvaz Jundishapur University of Medical Sciences. Data were analyzed using statistics, frequency distribution and Pearson’s correlation coefficient.

**Results:**

A total of 104,197 cases of scorpion stings was recorded from 2010 to 2015. The cumulative incidence of scorpion sting was 2.23%. The spatial distribution of scorpion stings showed that most cases occurred in the Dehdez district (4,504 scorpion stings/100,000 inhabitants) and the Masjed Soleyman county (4,069 scorpion stings/100,000 inhabitants). A significant association was found between climate factors (temperature, evaporation rate, sunshine duration, humidity, and precipitation) and the scorpion sting rate. An increase in rainfall and humidity coincided with a reduction in scorpion stings whereas an increase in temperature, evaporation, and sunshine duration was accompanied by a growth of scorpion stings. No significant correlation was found between wind velocity/direction and the incidence rate of stings. Moreover, the seasonal peak incidence of scorpion stings was recorded in summer (an average of 8,838 cases) and the lowest incidence was recorded during winter (an average of 1,286 cases). The annual trend of scorpion sting cases decreased during the period from 2010 to 2015.

**Conclusion:**

Climate variables can be a good index for predicting the incidence of scorpion stings in endemic regions. Since they occur mostly in the hot season, designing preventive measures in the counties and districts with a high incidence of scorpion stings such as Dehdez and Masjed Soleyman can minimize mortality and other burdens.

## Background

Scorpion sting is a major worldwide public health problem, especially in many tropical and subtropical countries including South India, Sahelian Africa, the Middle East, Mexico and South America [1, 2]. Global scorpion sting cases are estimated to be 1.2 million per year. Scorpion sting accounts for more than 3,250 fatalities (0.27%) [3]. Children are more prone to severe scorpion envenomation and their mortality rate is noticeable; however, adults are more generally concerned [3]. According to the World Health Organization (WHO) report, scorpion stings exert heavy psychological and socioeconomic impacts [4].

Scorpions (class Arachnida, order Scorpiones) belong to a major group of venomous arthropods. They have been observed in many habitats and can survive under severe conditions. This is thanks to their adaptive capacity that allows resistance to higher temperatures as well as water deprivation for a long period of time [5]. They also use the least energy. Scorpions use their stings to feed and defend. Some scorpion species make nests in the soil with smother patterns and proper physical structures. Thus, they are known as nest makers and diggers [6-8]. Some of them have adapted themselves to be active in or around human residential areas, thereby increasing the probability of their encounters with humans [9]. Scorpions are opportunistic predators concerning selecting their habitat and using any natural, artificial or human-made spaces and gaps for hiding and survival [10]. More than 2,200 scorpion species have been identified, of which about 25 species are potentially life threatening for humans [11].

Scorpion stings typically cause systemic and local manifestations. In most cases, the localized pain is the initial symptom. Itching, erythema, local swelling, and ascending hyperesthesia - that continues for more than a few weeks - are among the local signs of scorpion sting [12]. Systemic manifestations are induced by venom toxins that affect the ion channels (Na^+^, K^+^, Ca^2+^ and Cl^-^) and modify their functions [13, 14].

The severity of scorpion stings is influenced by two main variables: the victim characteristics (age, health condition) and the scorpion characteristics (species, venom potency) [15]. Various factors, including geographic location, regional socioeconomic structure, scorpion species, and climate conditions influence the prevalence of scorpion stings worldwide [16]. Children experience more severe envenomation [12, 17].

The incidence rates of scorpion stings vary in different geographic regions and countries [18]. The highest incidence rates were reported in Mexico and Iran, respectively [19]*.* Scorpion stings are a major public health problem in Iran (45,000-50,000 cases, 19 deaths annually) and neighboring countries (Iraq, Pakistan, Saudi Arabia, Oman, Yemen, and the United Arab Emirates) [20, 21].

In Iran, the scorpion distribution and species diversity are significant [18]. A total of 64 species of scorpions have been identified in Iran (distributed in 17 genera) and classified into three families: Buthidae (86%), Hemiscorpiidae (9.5%), and Scorpionidae (4.5%) [10]. *Androctonus crassicauda, Mesobuthus eupeus*, and *Hemiscorpius lepturus* are cited as the most dangerous species of Iranian scorpions and these species are responsible for most of the scorpion stings in the endemic area [15]. 

In Iran, the highest incidence of scorpion stings was reported in the Khuzestan and Hormozgan provinces [22, 23]. However, Khuzestan is ranked first in terms of scorpionism among the Iranian provinces [10, 24]. In Khuzestan (the southwestern province of Iran), the main causes of poisoning were attributed to scorpion stings (56%), drug poisoning (31%), and chemical exposure poisoning (5.5%) [25].

Due to the high prevalence of scorpionism in Khuzestan province, a demographic study of scorpionism and the factors associated with the increase in scorpionism in this region is necessary. The present study examined the relationship between different climate parameters (temperature, humidity, precipitation, evaporation, and wind) and the rate of scorpionism in Khuzestan province from April 2010 to March 2015.

## Methods

### Geographic and demographic characteristics of Khuzestan province

Khuzestan province with 29° 57’ up to 33° 0’ of the northern latitude of the equator and 47° 40’ up to 50° 33’ of the eastern longitude of the Greenwich Median is located in the south west of Iran. According to the country divisions in 2011, Khuzestan province has 24 counties and 62 cities. Ahvaz (the province capital) is the most populous county and Haftkel is the least populated county in Khuzestan province [26]. Khuzestan province with an area of 64,057 km^2^ has a population of 4,531,720 people (Population-Housing Census, 2011), 71.02% dwell in urban areas, 28.7% in rural areas, and the rest are non-residents [26].

### Demographics of scorpion stings

This research has studied all the cases of scorpion stings from various counties of Khuzestan province registered in the Department of Infectious Diseases, Ahvaz Jundishapur University of Medical Sciences, from April 2010 to March 2015. Clinical manifestations of the scorpion sting (redness around the sting site, local pain, numbness in the limb or body, and severe muscular pain) and systemic symptoms (signs of sympathetic/parasympathetic nervous systems, and central nervous system) were included in the study. The cumulative incidence rate for the scorpion stings in the province during the study period (2010-2015) was also calculated.

### Spatial distribution of scorpion stings in Khuzestan province (2010-2015)

**Figure 1.**
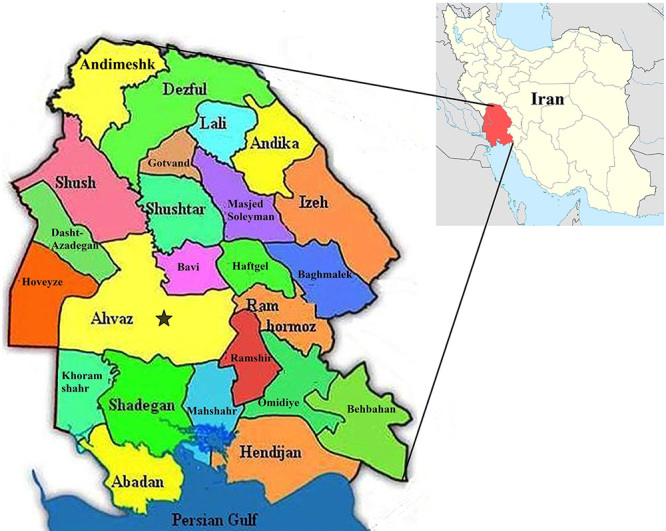
Map of Iran and the location of Khuzestan province.

In order to determine the spatial distribution of the scorpion sting, the population size and the frequency of scorpion stings in each city were obtained; and finally, the number of scorpion sting cases was calculated per 100,000 inhabitants (population statistics are included in Additional file 1, in the supplementary file). The spatial distribution map of scorpion sting cases in Khuzestan province is illustrated in Figure 1.

### Climate characteristics and determination method for Khuzestan province

Climate data (2010-2015) including average minima, maxima and mean annual air temperature (°C), humidity (%), precipitation (mm), evaporation (mm) and wind speed (km/h) by month and meteorological stations of each city were provided with the help of the Khuzestan Meteorological Offices.

Since the temperature and rainfall patterns were varying during the study period (a long period of below-freezing weather was reported during the study period), the De Martonne method was used to determine the type of climate of Khuzestan province during the study period (2010-2015) [27] (Table 1).

**Table 1.** Climate threshold based on De Martonne classification.

Climate type	Humidity indicator
Arid	<10
Semiarid	10-20
Mediterranean	20-24
Sub-humid	24-48
Wet	28-35
Very wet	>35

In the De Martonne method, the climate of a region is specified by calculating the ‘drought indicator’ (average annual precipitation divided by the average annual potential evapotranspiration (PET)) using the following aridity/humidity indicator:

$Aridity\;Index\;(I)=\frac{P}{T+10)}$ P: the average annual rainfall (mm), T: the normal rate of annual temperature (˚C).

### The relationship between climate factors and scorpionism (statistical analysis)

The mean seasonal values of climate data (average/minimum/maximum temperature, average/minimum/maximum humidity, total precipitation, the rate of evaporation, sunny hours, and wind) and the incidence of scorpion stings in the period of 2010-2015 were examined. Data were analyzed by applying descriptive statistics, frequency distributions and Pearson’s correlation coefficient. A p-value less than 0.01 was considered significant. All analyses were performed using SPSS version 21.

The reliability reported for the data of this study was measured adopting the test-retest with the Intraclass Correlation Coefficient (ICC) values ranging between 0.91 and 0.94. Validity was confirmed using the PEDro scale [28] by two independent reviewers, with disagreements resolved through consensus.

## Results

### Demographic analysis of scorpion stings

**Figure 2.**
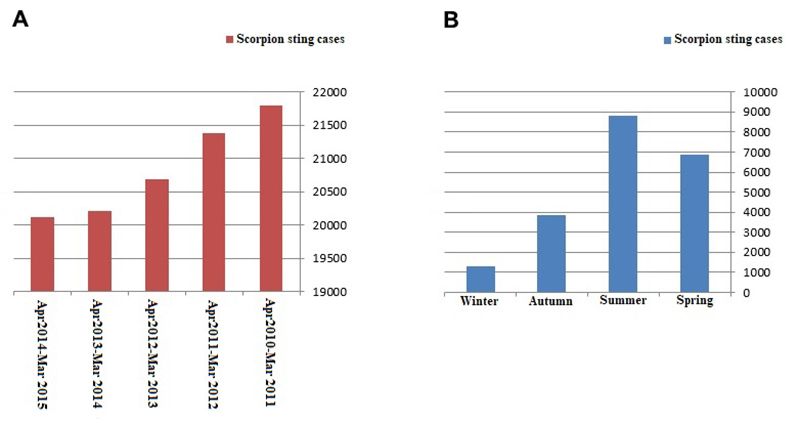
(A) The annual incidence of scorpion stings. (B) The mean seasonal incidence of scorpion stings in Khuzestan province, from April 2010 to March 2015.

A total of 104,197 cases of scorpion stings was recorded in Khuzestan province during the study period (2010-2015). The cumulative incidence rate of 2.3% for scorpion stings was obtained in the study period. The annual and seasonal frequency of scorpion stings are reported in Figure 2. The highest frequency of scorpion stings was observed from spring 2010 to winter 2011 (21,799 cases) while the lowest one was observed from spring 2014 to winter 2015 (20,120 cases) (Figure 2A). From the viewpoint of seasonal analysis, the highest frequency of scorpion stings was observed in the summer whereas the lowest one was observed in the winter (Figure 2B). It is worth mentioning that summer 2010 and winter 2013 experienced, the maximum and minimum sting cases among all other studied seasons, respectively.

The total number of scorpion sting cases for each city are reported in Additional file 2 in the supplementary file. The lowest frequency of scorpion sting (regardless of population) was reported in the Dehdez district (annual average = 73 cases) and the highest one was reported in Masjed Soleyman County (an annual average of 1,920 cases). The maximum coefficient of variation of frequency was observed in Shushtar County (196.8) and the lowest was observed in the Ramshir County (109.5).

### Analysis of climate parameters in Khuzestan province (2010-2015)

The annual values of the selected synoptic and climatological stations in Khuzestan province from April 2010 to March 2015 are shown in Additional file 3 in the supplementary file. The mean annual and monthly values of climate data over the period of the study are presented in Table 2.

**Table 2.** The annual and monthly values of climate factors in Khuzestan province (2010-2015).

Month parameter	April	May	June	July	August	September	October	November	December	January	February	March	Annual
Average temperature (°C)	22.6	28.8	24.3	36.6	37.3	42.2	28.7	21.3	14.8	12.6	13.9	16.7	25.15
Maximum temperature (°C)	29.3	36	42.8	45.8	45.7	42.7	36.9	27.6	22.5	18	19.3	22.9	32.4
Minimum temperature (°C)	15.7	21.8	26.2	28.6	29.2	25.8	20.7	15.1	9	7.2	8.6	10.8	18.2
Absolut maximum temperature (°C)	41.7	47	52	52.4	52.6	51.4	45.4	40.6	30.6	26.8	30	39.2	52.6
Absolut minimum temperature (°C)	8	6	9	9	15	15	5.8	2.4	-0.2	-4.8	-5.8	-3.8	-5.8
Sunlight hours	245.1	241.8	314.6	335.5	336.3	328	282.7	205.2	199.6	186	185.7	204.2	3064.7
Average humidity (%)	42.9	37.5	24	25.3	26.2	29.7	37.2	52.6	63.1	66.6	64.6	53.6	43.5
Average maximum humidity (%)	64.5	57.7	38.3	39.7	40.5	47.1	52.2	73	84.6	87.1	86.5	74.7	58.6
Average minimum humidity (%)	21.30	18.4	9.6	10.7	11.9	12.2	15.4	32.3	41.6	46.5	42.7	30.5	23.5
Maximum humidity (%)	100	100	100	100	100	100	100	100	100	100	100	100	100
Minimum humidity (%)	2	2	0	0.05	0.07	0.08	4	5	5	7	2	1	0
Rainfall (mm)	18.3	19.5	0.1	0	0.3	0.1	3	45.7	43.7	42.8	41.3	35.3	254.6
Evaporation (mm)	227.4	313.7	487.7	497.8	481.2	390.2	270.4	140.3	73.5	63.5	79.6	136.4	3161.7
Maximum wind velocity speed (km/s)	25	25	51	20	25	60	24	74	22	30	24	35	71
Maximum wind velocity direction (degree)	330	350	350	340	360	360	350	360	330	340	330	340	360

Temperature

**Figure 3.**
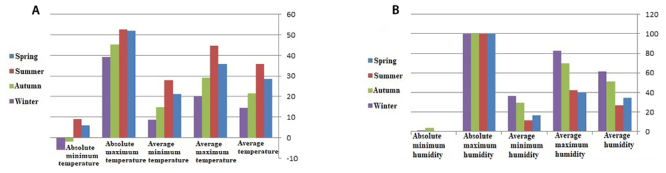
Temperature and relative humidity recorded in Khuzestan province, from April 2010 to March 2015. (A) Average seasonal temperature, average maximum and minimum seasonal temperatures, absolute maximum and minimum seasonal temperatures. (B) Average seasonal minimum/maximum relative humidity, absolute maximum and minimum seasonal relative humidity.

According to the recorded data, the minimum (coldest) temperature of the year was recorded in January (12.7 °C) whereas the maximum (hottest) temperature was recorded in August (37.3 °C) and July (36.6 °C) (Table 2). Likewise, during this study period, the mean seasonal temperatures were as follows: spring (28.5 °C), summer (38.63 °C), autumn (14.9 °C) and winter (14.4 °C) (Figure 3A). 

Humidity 

The average value of humidity was 43.3% during the study period. Furthermore, the most humid months were January and February, with an average relative humidity of 66.6% and 64.6%, respectively (Table 2). January and February exhibited the highest average monthly humidity values whereas June and July showed the lowest ones (Table 2). Overall, the most humidity occurred in winter and the least was observed in summer (Figure 3B).

Sunshine

**Figure 4.**
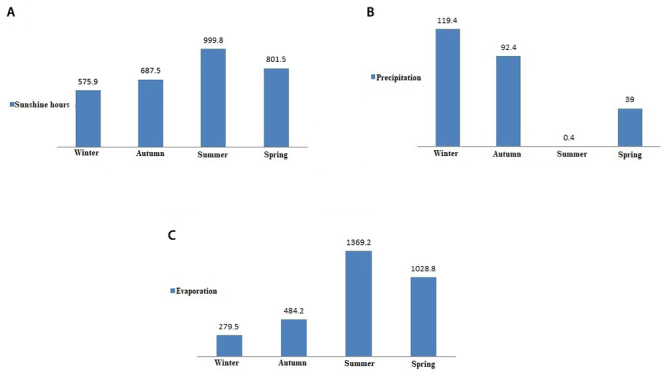
Average climate data recorded in Khuzestan province, from April 2010 to March 2015: (A) seasonal sunshine hours, (B) seasonal rainfall, and (C) seasonal evaporation.

With regard to the results detailed in Table 2, Khuzestan province showed a trend of long sunshine hours between April and September. The maximum sunshine hours were observed in August (336.3 hours) and the minimum sunshine hours were observed in January (186 hours) and February (185.7 hours). The maximum seasonal sunshine hours were 999.8 hours in summer, 801.5 hours in spring, 687.5 hours in autumn, and 575.9 hours in winter (Figure 4A).

Precipitation 

The highest monthly average of precipitation over the study period was recorded in November (45.7mm), December (43.7mm) and January (42.8mm) (Table 2). The highest seasonal mean value of precipitation was recorded in winter (119.4mm) and autumn (92.4mm), but on the other hand, the lowest one was recorded in spring (37.9mm). Summer was the driest season of the year (0.4mm) (Figure 4B).

Evaporation 

The total evaporation rates in the Khuzestan climate models were high over the period of the study (ranged from 63.5 mm in January to 497.8 in July). In fact, with the onset of January, the uptrend of evaporation began to rise, culminating in July and then declining to its lowest level in January (Table 2). Summer showed the utmost evaporation rate with 1,369.2 mm whereas winter showed the slightest one with 279.5 mm (Figure 4C).

Wind velocity 

The highest wind speed was recorded in November (74 km/hr.) while the lowest one was recorded in July (20 km/h). The average wind speed in the warmer months of the year was greater than the colder months (Table 2). The wind direction at different meteorological stations was varied between 310 and 360° (Additional file 3).

Determination of climate type 

The average annual temperature of Khuzestan province in the study period was 25.15 °C and the average annual rainfall was 254.6 mm. According to the De Martonne climate classification, Khuzestan can be viewed as a Mediterranean climate region with a threshold of humidity index (HI) equal to 20.27 (Table 3).

**Table 3.** Climate threshold in the meteorological stations of Khuzestan province based on De Martonne classification, from April 2010 to March 2015.

Station	Moister index threshold	Climate threshold
Omidieh	17.5	Semiarid
Ahvaz	16.4	Semiarid
Izeh	33.3	Wet
Abadan	13.5	Semiarid
Bostan	18.3	Semiarid
Behbahan	23.2	Mediterranean
Dezful	20.9	Mediterranean
Dehdez	32.5	Wet
Ramhormoz	17.4	Semiarid
Shadegan	13.9	Semiarid
Shooshtar	19.8	Semiarid
Shoosh	17.8	Semiarid
Gotvand	19.9	Semiarid
Lali	23.9	Mediterranean
Mahshahr	15.6	Semiarid
Masjed-Soleyman	22.9	Mediterranean
Hendijan	17.8	Semiarid
Total	20.27	Mediterranean

### Association between climate and scorpion sting cases

The coefficients (r) of the correlation between scorpion sting cases and climate factors are presented in Table 4. No significant correlation was observed between wind velocity and incidence of scorpion stings (p-value > 0.01). A significant positive correlation was found between scorpion sting cases and climate factors, including temperature, evaporation, and sunny hours (p-value < 0.01). As temperature, evaporation, and sunny hours increased the incidence of scorpion stings rose, accordingly. However, a negative correlation was found between the incidence of the scorpion sting, and relative humidity and precipitation (p-value < 0.01); so, as humidity and precipitation increased, the incidence of scorpion stings decreased. Pearson correlation coefficients between weather variables - temperature (0.977), average humidity (-0.943), rainfall (-0.807), evaporation (0.970), and sunlight hours (0.967) - and incidence of scorpion sting indicated that, among the investigated variables, temperature was more correlated with incidence of scorpion sting.

**Table 4.** The correlation and significant level of seasonal variations of climate variables and scorpion sting cases, April 2010-March 2015.

Parameter	Pearson correlation coefficient (r)	p-value
Maximum temperature	0.923	0.000
Minimum temperature	0.869	0.000
Average temperature	0.977	.000
Average maximum temperature	0.978	0.000
Average minimum temperature	0.971	0.000
Maximum humidity	-0.713	0.000
Minimum humidity	-0.799	0.000
Average humidity	-0.943	0.000
Average maximum humidity	-0.955	0.000
Average minimum humidity	-0.937	0.000
Wind	-0.341	0.141
Rainfall	-0.807	0.000
Evaporation	0.970	0.000
Sunlight hours	0.967	0.000

### Spatial distribution of scorpion stings in the Khuzestan province

**Figure 5.**
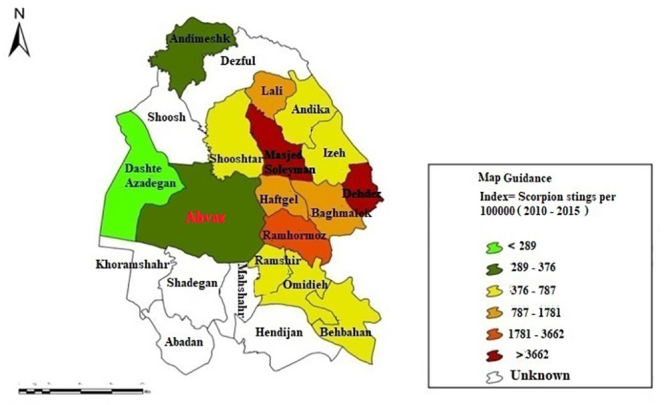
Spatial distribution map of scorpion sting cases in Khuzestan province, from April 2010 to March 2015.

The spatial distribution map and the frequency of scorpion stings in Khuzestan province are shown in Figure 5. The highest incidence of scorpion stings was reported in the Dehdez district (4,504 scorpion stings per 100,000 inhabitants) and Masjed Soleyman County (4,069 scorpion stings per 100,000 inhabitants). The lowest incidence of scorpion stings was reported in the counties of Andimeshk (375 scorpion stings per 100,000 inhabitants), Ahvaz (312 scorpion stings per 100,000 inhabitants), and Dasht-e Azadegan (288 scorpion stings per 100,000 inhabitants), respectively. Ramhormoz, Lali, Baghmalek, Haftkel, Behbahan, Izeh, Ramshir, Omidiyeh, Shushtar, and Andika are in the subsequent ranks of the scorpion sting in descending order.

## Discussion

Various factors such as the geographical location, social and economic structure of a region, scorpion species, and the climate conditions are involved in the incidence of scorpion sting in different geographical regions [16]. The latter (climate conditions) has not been much studied and should be properly examined. The present study explored the relationship between different climate parameters (temperature, sunny hours, humidity, precipitation, evaporation, and wind) and the rate of scorpionism in Khuzestan province of Iran. The results of the present study revealed a direct relationship between the first five aforementioned climate parameters and the incidence of the scorpion sting; therefore, with a rise in temperature, evaporation, and sunny hours, the incidence of scorpion stings went up, while with an increase in the humidity and precipitation, the incidence of scorpion stings went down. It should be noted that no significant correlations were found between wind direction, wind velocity, and incidence of scorpion sting. Furthermore, it was realized that summer had the highest prevalence of scorpionism and winter had the lowest one in Khuzestan province over the study period. The results of the study also demonstrated a declining annual trend of scorpionism. Spatial distribution for scorpion sting indicated that the Dehdez district and Mahshahr County had the highest prevalence of scorpion sting in Khuzestan province.

In Iran, Khuzestan province is highlighted for its highest incidence of scorpion stings [29]. In a study published in 2012, Kassiri et al. [30] showed that the scorpion sting was the leading cause of poisoning among non-medicinal poisoning agents in Khuzestan province. Similarly, in another work, Dehghani et al. [29] concluded that 94,448 scorpion sting cases (more than 55%) in Iran (2001- 2005) were related to Khuzestan province. In the present study, the cumulative incidence of scorpion stings was 2.3%, while in the one by Dehghani et al. [29] this rate was calculated to be 2.2%.

Dehghani et al. [29] maintained that there was no definite trend for scorpion stings; however, in the present study, a declining annual trend of scorpion stings in the study period has been suggested. In the present study, the highest (21,799 cases) and lowest (20,120 cases) incidence of scorpion stings were recorded in 2010 and 2015, respectively (Figure 2A). The reason could be explained through such factors as people’s awareness, preventive measures, development of health care facilities, improvement in self-care behaviors and higher levels of housing security and construction materials.

According to the De Martonne climate classification, Khuzestan was classified as Mediterranean. Similarly, various studies have reported the prevalence of scorpion stings in the Mediterranean areas [16, 31]. In a study in 2019, Abd El-Aziz et al. [31] concluded that scorpion sting is particularly health-threatening in Luxor (Southern Egypt, Mediterranean climate zone). A total of 110 cases of scorpion stings was reported in 2017. In the same way, Turkey with the Mediterranean climate zone is pointed up for the incidence of scorpion stings [32]. In a study in 2008, Ozkan et al. [16] reported 24,261 scorpion sting cases in Turkey in 2005. Consequently, a close association was proved between prevalence of scorpion stings and the Mediterranean climate zone.

Spatial distribution analysis of scorpion stings in Khuzestan province revealed that the Dehdez district and Masjed Soleyman County had the highest incidence of scorpion stings; on the other hand, Andimeshk, Ahvaz, and Dasht-e Azadegan Counties exhibited the lowest incidence of scorpion stings (Figure 5). In a study in 2014, Kassiri et al. [33] concluded that scorpion stings represent a public health challenge in Masjed Soleyman County. 

The effects of some different climate factors on the prevalence of scorpion stings in some of the Khuzestan province counties such as Dezful, Ramshir, and Baghmalek were reported in the previous studies [34-36]. Nonetheless, in the present study, all climate variables of the counties of Khuzestan province have been comprehensively taken into account. The highest and lowest seasonal incidence of scorpion stings were, respectively, observed in summer and winter. 

Ebrahimi et al. [37] in 2017 focused the relationship between climate factors and the prevalence of scorpionism in Haji-Abad (the north of Hormozgan province, in the south of Iran). The results of the study reflected that temperature (positively) and relative humidity (negatively) were associated with the incidence of scorpion sting cases [37].

In a study published in 2005, Chowell et al. [38] examined the association between the incidence of scorpion stings and several climate variables in state of Colima (Mexico) in 2000-2001 period and concluded that the number of scorpion stings was independent of actual rainfall when the rainfall >30 mm/month. The results of their study showed no association between the rate of evaporation and the prevalence of scorpion stings, which is inconsistent with outcome of the present research. Nevertheless, similar to the results of the present study, they found out that as temperature increased, the incidence rate of scorpion stings increased, in turn [38]. In 2014, Selmane and L’hadj [39] studied the relationship between scorpion sting cases and the climate conditions in M’Sila province (Algeria) from 2001 to 2010 and inferred that temperature had a direct bearing on the incidence of scorpion stings, which is consistent with our results. In the same way, most scorpion sting cases were observed during the summer period in Brazil [40], Egypt [41] and Morocco [42] compared to other months of the year, confirming results of the present study. The highest incidence of scorpion stings was recorded in summer (the highest recorded temperature), which proves the correlation between temperature and incidence of scorpion stings.

The redness at the spot of a scorpion sting and local pain can be a sign and symptom of it. Even so, this symptom can be found in any arthropod stings. This could be the limitation of this study. Having said that, the combination of the above symptoms with numbness in the limb or body, severe muscular pain, and systemic symptoms can increase the certainty about scorpion stings. It is worth mentioning that in some cases envenomed people brought the scorpion that had stung them to the medical center.

## Conclusion

Climate variables can be a good index for the prediction of scorpion stings incidence in endemic regions. Since scorpion stings occur mostly in the hot season, designing preventive measures in counties and districts with the high incidence of scorpion stings such as Dehdez, Masjed Soleyman, can minimize the mortality and other burdens, especially in the hot season.

## Supplementary material

The following online material is available for this article:

Additional file 1. Population statistics of different cities of Khuzestan province (https://www.amar.org.ir/).

Additional file 2. The total number of scorpion sting cases in Khuzestan province (2010-2015).

Additional file 3. Annual climate variability and change at the selected synoptic stations in Khuzestan province, from April 2010 to March 2015.
