# Supplementary material for: Effects of climate variables on the incidence of scorpion stings in Iran for five years
Source: J Venom Anim Toxins Incl Trop Dis. 2021 Jun 30;27:e20200110. doi: 10.1590/1678-9199-JVATITD-2020-0110 (PMC8252957; doi:10.1590/1678-9199-JVATITD-2020-0110)
Supplement: Supplementary file 1 [file 1678-9199-jvatitd-27-e20200110-s1.pdf]

J Venom Anim Toxins incl Trop Dis

https://doi.org/10.1590/1678-9199-JVATITD-2020-0110

Supplementary Material to "Effects of climate variables on the incidence of scorpion stings in Iran for five years"

Additional file 1. Population statistics of different cities of Khuzestan province (https://www.amar.org.ir/).

City	Total population	Women	Men	Rural areas	Urban points	Non-resident
Abadan	271484	135916	135568	42579	228905	
Omidieh	90420	44911	45509	27558	62862	
Andika	50797	25002	25795	48431	2272	94
Andimeshk	167126	81212	85914	34985	128774	3367
Ahvaz	1484344	737110	747234	296455	1187889	
Izeh	203621	102510	101111	81581	122013	27
Baghmalek	107450	53840	53610	63808	43582	60
Bandare mahshahr	278037	137794	140243	20764	257273	
Behbahan	179703	89112	90591	53270	126304	129
Khoramshahr	163701	82826	80875	32994	130707	
Dezful	423552	204641	218911	131465	296979	4744
Dashte-Azadegan	99831	49793	50038	48104	51727	
Ramshir	48943	24226	24717	24142	24801	
Ramhormoz	105418	52317	53101	35013	69869	536
Shadegan	153355	76394	76961	94810	58545	
Shoosh	202762	100729	102033	115028	86976	758
Shooshtar	191444	93932	97512	70317	120687	440
Gotvand	64951	31629	33322	20238	43881	832
Lali	37381	18331	19050	19454	17745	182
Masjed-Soleyman	113257	56474	56783	9149	103369	739
Haftgel	22391	11207	11184	7421	14877	93
Hendijan	37440	18742	18698	9234	28206	
Hoveizeh	34312	16863	17449	14468	19844	
Total of Province	4531720	2245511	2286209	1301268	3228087	12001
