# Supplementary material for: Effects of climate variables on the incidence of scorpion stings in Iran for five years
Source: J Venom Anim Toxins Incl Trop Dis. 2021 Jun 30;27:e20200110. doi: 10.1590/1678-9199-JVATITD-2020-0110 (PMC8252957; doi:10.1590/1678-9199-JVATITD-2020-0110)
Supplement: Supplementary file 2 [file 1678-9199-jvatitd-27-e20200110-s2.pdf]

J Venom Anim Toxins incl Trop Dis

https://doi.org/10.1590/1678-9199-JVATITD-2020-0110

Supplementary Material to “Effects of climate variables on the incidence of scorpion stings in Iran for five years”

Additional file 2. The total number of scorpion sting cases in Khuzestan province (2010-2015).

City	Annual average	Minimum	Maximum	Coefficient of variation (CV)
Andika	272	23	486	154.4
Andimeshk	141	15	253	180.2
Omidieh	97	10	214	131.4
Ahvaz	906	53	2207	134.7
Izeh	651	52	1340	141.1
Baghmalek	574	26	1430	116.7
Behbahan	588	64	1230	146.4
Dashte Azadegan	120	13	307	119.8
Dehdez	73	6	143	143.4
Ramhormoz	1608	166	3350	135.3
Shooshtar	363	69	625	196.8
Lali	277	46	620	160
Masjed-Soleyman	1920	272	3046	194.6
Haftgel	92	12	205	125.7
Ramshir	93	3	273	109.5
