# Supplementary material for: Effects of climate variables on the incidence of scorpion stings in Iran for five years
Source: J Venom Anim Toxins Incl Trop Dis. 2021 Jun 30;27:e20200110. doi: 10.1590/1678-9199-JVATITD-2020-0110 (PMC8252957; doi:10.1590/1678-9199-JVATITD-2020-0110)
Supplement: Supplementary file 3 [file 1678-9199-jvatitd-27-e20200110-s3.pdf]

J Venom Anim Toxins incl Trop Dis

https://doi.org/10.1590/1678-9199-JVATITD-2020-0110

Supplementary Material to "Effects of climate variables on the incidence of scorpion stings in Iran for five years"

Additional file 3. Annual climate variability and change at the selected synoptic stations in Khuzestan province, from April 2010 to March 2015.

Parameter station	Average temperature (°C)	Minimum temperature (°C)	Maximum temperature (°C)	Average humidity (%)	Average maximum humidity (%)	Average minimum humidity (%)	Evaporation (mm)	Rainfall (mm)	Sunlight hours	Maximum wind velocity speed (km/s)	Maximum wind velocity direction (degree)
**Omidieh**	24.4	-2.4	52.6	42.7	61.8	23.7	3565.1	200.5	3120.1	25	340
**Ahvaz**	26.4	-1.2	51.6	44.9	64.8	25.2	3222.7	169.3	3155	17	350
**Izeh**	21.4	-5.8	46.6	40.8	59.3	22.7	2387.8	499.8	3086.1	16	310
**Abadan**	26.79	-2.2	52	43.98	65.18	23	3888.9	94.3	3140.3	21	360
**Bostan**	25.3	-3.8	51.6	50.8	66.1	24.7	3626.6	208.9	3040.8	25	320
**Behbahan**	24.8	-3.2	50.6	43.4	64.9	23.4	3389.2	328.3	3176.5	25	320
**Dezful**	24.7	-2.6	51.8	49.9	71.3	27.4	2489.1	272.5	2944.7	25	330
**Shooshtar**	26.9	-1	52.6	40.5	53.6	27.4	3001	265.7	3052.6	13	350
**Mahshahr**	26.3	-2	51.4	47.9	69	26.8	3752.4	148.5	3155.7	20	350
**Ramhormoz**	27.1	-1.8	52	35.9	50.8	21.1	3065.6	202.6	3155.1	12	330
**Hendijan**	26.1	-2.4	52	43	62.2	23.5	3448.1	204.2	3312.4	25	350
**Masjed Soleyman**	25.9	-3.6	52	38.9	53.9	23.1	3303.7	331.8	2922.4	22	340
**Shadegan**	26.1	-0.4	50.2	44	65.2	23	3888.9	102.2	2999.1	22	360
**Lali**	24.6	-4	50.6	38.5	54.1	23	3295.8	343.2	2976.1	20	330
**Dehdez**	18.6	-5.6	41.8	40.7	59.3	22.4	2365.1	418.6	3246.6	13	310
**Shoosh**	25.3	-1.8	52	49.2	70.6	26.7	2503	198.4	2938.2	60	340
**Gotvand**	24.6	-1.6	53	40.4	35.5	27.4	2997.7	244.9	3004.3	17	360
**Andimeshk**	26.3	-1	50.6	48.8	70.9	26.1	2535.8	285.5	2816.6	74	330
**Aghajari**	24.2	-0.6	51.12	44.1	64.9	23.9	3357.2	230.5	2988	25	330
